# Pituitary tumors and the risk of other malignancies: is the relationship coincidental or causal?

**DOI:** 10.1530/EO-21-0033

**Published:** 2022-12-22

**Authors:** Sandra Pekic, Marko Stojanovic, Vera Popovic

**Affiliations:** 1 School of Medicine, University of Belgrade, Belgrade, Serbia; 2 Clinic for Endocrinology, Diabetes and Metabolic Diseases, University Clinical Center Belgrade, Belgrade, Serbia

**Keywords:** pituitary tumor, malignancy, family history, endocrine disruptor

## Abstract

Pituitary adenomas are benign neoplasms of the pituitary. The most prevalent are prolactinomas and non-functioning pituitary adenomas, followed by growth hormone- and ACTH-secreting adenomas. Most pituitary adenomas seem to be sporadic and their persistent growth is very atypical. No molecular markers predict their behavior. The occurrence of pituitary adenomas and malignancies in the same patient can be either pure coincidence or caused by shared underlying genetic susceptibility involved in tumorigenesis. Detailed family history on cancers/tumors in the first, second and third generation of family members on each side of the family has been reported in a few studies. They found an association of pituitary tumors with positive family history for breast, lung and colorectal cancer. We have reported that in about 50% of patients with pituitary adenomas, an association with positive family history for cancer has been found independent of secretory phenotype (acromegaly, prolactinoma, Cushingʼs disease or non-functioning pituitary adenomas). We also found earlier onset of pituitary tumors (younger age at diagnosis of pituitary tumors) in patients with a strong family history of cancer. In our recent unpublished series of 1300 patients with pituitary adenomas, 6.8% of patients were diagnosed with malignancy. The latency period between the diagnosis of pituitary adenoma and cancer was variable, and in 33% of patients, it was longer than 5 years. Besides the inherited trophic mechanisms (shared underlying genetic variants), the potential influence of shared complex epigenetic influences (environmental and behavioral factors – obesity, smoking, alcohol intake and insulin resistance) is discussed. Further studies are needed to better understand if patients with pituitary adenomas are at increased risk for cancer.

## Introduction

Pituitary adenomas are usually benign neoplasms arising from anterior pituitary cells, grow slowly and usually change little in size over many years. The autopsy and MRI studies showed that 10–15% of patients with no history of pituitary disease had previously undiagnosed pituitary tumors, incidentalomas and clinically relevant pituitary tumors (Hall *et al.* 1994, Burman & Saeger 2006). Epidemiological studies show an increased incidence and prevalence of pituitary tumors in the general population, with approximately 1 case per 1000 of the general population (Daly & Beckers 2020). The most prevalent are prolactin-secreting adenomas (50–60%), non-functioning pituitary adenomas (20–40%), growth hormone-secreting adenomas (10–15%) and adrenocorticotropin-secreting adenomas (5–8%) (Molitch 2017, Daly & Beckers 2020, Melmed 2020, Ho *et al.* 2021). In summary, pituitary adenomas are common in the general population, majority are benign tumors and they change little in size over many years. Recently it has been shown that non-functioning microadenomas (<1 cm in size) have a mean and median time to growth of 38.1 and 24.5 months, respectively (Han *et al.* 2021). A small proportion of pituitary tumors behaves aggressively, that is affect the surrounding structures.

The number of genetic factors linked to pituitary tumors is very small. Usually, pituitary tumors may be diagnosed as a part of hereditary endocrine neoplasia, such as multiple endocrine neoplasia types 1 and 4, familial isolated pituitary adenomas, succinate dehydrogenase mutations (*SDHA*, *SDHB*, *SDHC*, *SDHD*, *MAX*, *TMEM 127*) and other conditions (Mc Cune Albright Sy, Carney complex, neurofibromatosis type 1, von Hippel–Lindau syndrome and tuberous sclerosis complex (TSC1 and TSC2). In this review, we will not discuss these hereditary syndromes.

Here, we will discuss risk factors for tumorigenesis for both pituitary tumors and cancers. We will not discuss the role of hormones in the progression of cancers. It is known that hormones stimulate the proliferation of cancer cells and promote tumors growth but are not carcinogens.

## The risk of malignancy in patients with sporadic pituitary adenomas

It is still a matter of debate whether patients with pituitary adenomas are at increased risk of malignant tumors (Popovic *et al.* 1998, Boguszewski & Ayuk 2016, Olsson *et al.* 2017). The incidence of malignancy was studied in patients with chronic excess of growth hormone (acromegaly) in particular, while this topic has been rarely studied in patients with non-functioning pituitary adenoma and prolactinomas (Popovic *et al.* 1998, Erfurth *et al.* 2001, Minniti *et al.* 2005, Norberg *et al.* 2008, Sattler *et al.* 2012, Child *et al.* 2015, Boguszewski & Ayuk 2016, Olsson *et al.* 2017, Terzolo *et al.* 2017, Battistone *et al.* 2021).

### Acromegaly and risk for malignancy

This association of growth hormone (GH)/insulin-like growth factor 1 (IGF-1) axis and cancer risk is complex. Experimental studies showed that GH and IGFs have pro-mitogenic and anti-apoptotic properties and have an important role in growth, metabolism, control of cell cycle and chemoresistance (Seccareccia & Brodt 2012, Strous *et al.* 2020, Kasprzak 2021). Experimental studies show that GH is permissive for neoplastic colon growth and induces colon DNA damage independent of IGF-1 (Chesnokova *et al.* 2016, Chesnokova *et al.* 2019). Growth hormone mediates the colon microenvironment by suppressing tumor-suppressor genes, such as p53 and adenomatous polyposis coli (APC) in colon cells, with subsequent decreased p21 expression and apoptosis (Chesnokova *et al.* 2016). GH excess enables cell survival and motility, while administration of a GH receptor blocker (pegvisomant) induces these tumor-suppressor genes (p53 and APC) (Chesnokova *et al.* 2016). GH in colon cells also decreases DNA repair and increases DNA damage accumulation by direct effect, independently of IGF-1, thus promoting chromosomal instability (Chesnokova *et al.* 2019). Overall although GH may have a role in the progression of malignancy, it does not induce malignancy.

Many studies showed that high-normal serum IGF-1 levels may be associated with an increased risk of malignancy in the general population (Pollak *et al.* 2004, Renehan *et al.* 2004, Samani *et al.* 2007, Murphy *et al.* 2020, Watts *et al.* 2021). Also, studies on hypopituitary experimental animals (dwarf mice, GH receptor/BP knockout mice) showed a lower incidence of malignancy and increased longevity (Bartke & Brown-Borg 2004, Ikeno *et al.* 2009, Bartke *et al.* 2016, Duran-Ortiz *et al.* 2021). Similar results were shown in some patients with congenital hypopituitarism due to PROP-1 mutation (few people of Krk) or congenital IGF-1 deficiency (Shevah & Laron 2007, Krzisnik *et al.* 2010, Guevara-Aguirre *et al.* 2011). However, cases from the literature illustrate that even severe isolated GH deficiency does not protect completely from the development of malignancy (Aguiar-Oliveira *et al.* 2010). We reported a 42-year-old female with combined familial congenital hypopituitarism (PROP1 mutation) and severe GH deficiency, who died from cancerous leptomeningitis due to advanced ovarian cancer (Vujovic *et al.* 2016). She had six close relatives with malignant disease. We also managed a 71-year-old male patient with congenital hypopituitarism with lifelong untreated GH deficiency, who died from disseminated renal cancer. His sister had a history of breast cancer. Both of our patients with GH deficiency had family history of malignancy, the risk factor which we will later discuss in more detail.

In the clinical settings of chronic supra-physiological growth hormone and IGF-1 levels in patients with acromegaly, the risk to development of malignancies is still controversial (Loeper & Ezzat 2008, Boguszewski & Ayuk 2016, Boguszewski & Boguszewski 2019). Cancer-related mortality in active acromegalic patients shows conflicting results. In some studies, mortality is increased, while in others, it is not (Orme *et al.* 1998, Popovic *et al.* 1998, Holdaway *et al.* 2004, Kauppinen-Makelin *et al.* 2005, Sherlock *et al.* 2009, Colao *et al.* 2014, Terzolo *et al.* 2017, Esposito *et al.* 2018, 2021). Large epidemiological and observational studies on all cancers, colorectal cancer and thyroid cancers show inconsistent results (Orme *et al.* 1998, Jenkins & Besser 2001, Gullu *et al.* 2010, Battistone *et al.* 2021).

A large nationwide population-based study from Sweden investigated the incidence of benign and malignant tumors in 1296 patients with acromegaly (Esposito *et al.* 2021). The authors reported increased risk of both benign (more than two-fold) and malignant tumors (by 30%), especially colorectal and anal cancer (50% higher), as well as kidney and ureteral cancer (four-fold higher) in acromegaly in comparison with the Swedish general population. The incidence of thyroid cancer was not increased in this study (only 3 cases were recorded in 1296 acromegalics). Age at acromegaly diagnosis was significantly related to increased cancer risk, while gender, hypopituitarism or diabetes mellitus were not associated with cancer risk (Esposito *et al.* 2021).

An interesting pilot study investigated the possible protective role of metformin on the development of colonic polyps in 58 patients with acromegaly (Albertelli *et al.* 2021). A significant negative association between metformin intake and colonic polyps was demonstrated: 57% of patients without polyps were treated with metformin, while only 24% of patients with polyps (including three adenocarcinomas) were treated with metformin. The results of this study could suggest a protective role of metformin but need to be confirmed in larger studies.

As for differentiated thyroid cancer in patients with acromegaly, there is some evidence for the role of genetic events for the onset of thyroid cancer with no correlation with disease activity or GH/IGF-1 levels. Mian *et al*. proposed that risk for thyroid cancer might be associated with BRAF mutations and aryl hydrocarbon receptor (AhR) overexpression (Mian *et al.* 2014). BRAF V600E was found in 70% of the papillary thyroid cancers, and AhR-interacting protein (AIP) was expressed more in papillary thyroid cancers particularly carrying BRAF mutations than in normal tissue, irrespective of acromegaly activity (Mian *et al.* 2014). The acromegalic patients with differentiated thyroid cancer were older and more often female, with similar levels of GH/IGF-1 compared with acromegalic patients without thyroid cancer (Mian *et al.* 2014). Other studies showed much lower prevalence of BRAF V600E mutations (14.3%) and NRAS mutations (21.4%) in acromegalic patients with thyroid cancer (Aydin *et al.* 2016). The impact of these mutations in promoting thyroid carcinoma in acromegaly remains to be defined in further studies. Current data do not recommend screening for thyroid cancer at diagnosis of acromegaly. In those patients with palpable thyroid nodule and other risk factors for thyroid cancer, the recommendations are the same as for the general population (Giustina *et al.* 2020).

The Italian nationwide multicenter cohort study of 1512 acromegalic patients showed that acromegaly was associated with a moderate increase in cancer risk for all cancers. Multivariate analysis showed that the increased risk of malignancy was not associated with GH/IGF-1 concentrations or duration of acromegaly (non-significant association with duration of acromegaly), but it is associated with age and family history of cancer (Terzolo *et al.* 2017). Colonoscopy screening in patients with acromegaly at diagnosis showed an increased risk of preneoplastic colonic lesions and colorectal cancer in these patients, compared with the general population, with insulin resistance as a risk factor for colonic polyps (Battistone *et al.* 2021). Recently, recommendations for screening thyroid and colorectal cancers in patients with acromegaly have been published (Terzolo *et al.* 2020).

Cancer becomes a leading cause of death in acromegalic patients with improved life expectancy (Arosio *et al.* 2012, Bogazzi *et al.* 2013, Mercado *et al.* 2014, Ritvonen *et al.* 2016, Maione *et al.* 2017, Bolfi *et al.* 2018). The cancer type includes different malignancies that are not traditionally related to acromegaly (such as colon and thyroid cancer): anaplastic thyroid carcinoma, glioblastoma, ovarian adenocarcinoma, uterine, lung, liver, prostate, breast, ovary, brain, gastric, pancreatic, hematological and melanoma (Orme *et al.* 1998, Holdaway *et al.* 2004, Kauppinen-Makelin *et al.* 2005, Sherlock *et al*. 2009, Colao *et al.* 2014, Bolfi *et al.* 2018, Esposito *et al.* 2018). These different cancer types are typically associated with age, genetic and environmental factors (Bolfi *et al.* 2018).

Recently, experts in the management of acromegaly published recommendations regarding comorbidities in acromegaly (Giustina *et al.* 2020). It is suggested that acromegaly patients undergo screening colonoscopy at diagnosis, but there are no conclusive data about screening frequency (Giustina *et al.* 2020). There are some difficulties in cancer diagnosis in patients with acromegaly. Colonoscopy can be technically challenging in these patients due to dolichocolon and tortuous bowel loops and should be performed by an experienced gastroenterologist (Gadelha *et al.* 2019). Also, only 25% of patients had a colonoscopy at diagnosis of acromegaly, while others had first colonoscopy after a mean of 9 years after acromegaly diagnosis (Parolin *et al.* 2018). It remains unknown if this increased incidence of some types of malignancy in acromegaly (colorectal cancer) is due to more screening (Gadelha *et al.* 2019).

### Non-functioning pituitary adenoma and risk for malignancy

The incidence of malignancy in patients with non-functioning pituitary adenoma has been sparsely studied (Popovic *et al.* 1998, Olsson *et al.* 2017, Hammarstrand *et al.* 2018). Previous analysis of our database with 469 patients with pituitary adenoma showed that the overall incidence of malignancy in patients with acromegaly and non-functioning pituitary adenomas was significantly increased (Table 1) (Popovic *et al.* 1998). In our recent unpublished larger series of 1300 patients with pituitary adenomas, 88 patients (6.8%) were diagnosed with malignancy, almost half of them (*n*  = 43) had non-functioning pituitary adenoma. Clinical characteristics and type of malignancy in these patients are presented in Table 2. The latency period between the diagnosis of pituitary adenoma and cancer was variable, and in 33% of patients, it was longer than 5 years. These data are in accordance with the large study of the Swedish Cancer Register (Olsson *et al.* 2017). These authors demonstrated that patients with non-functioning pituitary adenoma (*n*  = 2795) have an increased overall risk of developing malignancies (only men) and increased incidence of neoplasms of the brain, skin and melanoma (Olsson *et al.* 2017). Another study of 426 patients with non-functioning pituitary adenoma did not confirm increased incidence of malignant tumors, either in patients with or without GH replacement therapy (Hammarstrand *et al.* 2018).

**Table 1:** Frequency of cancer in patients with non-functioning pituitary tumour, prolactinoma and acromegaly compared with the general population (GP) and with the internal control group (Graves’ disease–GD). Reproduced with permission from John Wiley and Sons Ltd from Popovic *et al.* (1998). © 1998.

Tumor	Number of patients	Patient-years	Observed	Malignancy		SIR-GP (95% CI)	SIR-GD (95% CI)
				Expected (GP)	Expected (GD)		
Non-functioning							
All	151	609	11	2.56	2.70	3.91(1.88–7.19)^a^	4.07 (2.03–7.29)^a^
Female	86	371	4	1.19	1.29	2.52 (0.52–7.36)	3.10 (0.84–7.94)
Male	65	238	7	1.26	2.31	5.56 (2.·23–11.45)^a^	3.03 (1.22–6.24)^a^
Prolactinoma							
All	98	522	2	2.19	2.32	0.91 (0.11–3.29)	0.86 (0.10–3.10)
Female	67	366	2	1.17	1.28	1.71 (0.21–6.17)	1.56 (0.19–5.64)
Male	31	156	0	0.83	1.51	0 –	0 –
Acromegaly							
All	220	1546	23	6.49	6.86	3.39 (2.12–5.12)^a^	3.21 (2.01–4.85)^a^
Female	137	936	14	2.99	3.26	4.35 (2.31–7.44)^a^	3.99 (2.12–6.82)^a^
Male	83	610	9	3.23	5.92	2.78 (1.27–5.28)^a^	1.52 (0.70–2.89)

^a^*P*< 0.01.

**Table 2:** Clinical characteristics and type of malignancy in patients with pituitary adenoma.

	Non-functioning PA	Acromegaly	Prolactinoma	Morbus Cushing	Total
*n*	43	21	20	4	88
Sex (F/M)	26/17	12/9	10/10	1/3	49/39
Age at PA diagnosis (X ± s.e.)	55.2 **±** 2.2	48.3 ± 2.9	49.1 ± 2.8	49.0 ± 7.0	51.9 ± 1.5
Age at Mg diagnosis (X ± s.e.)	53.5 ± 2.3	53.2 ± 3.2	50.4 ± 3.2	58.8 ± 8.1	53.0 ± 1.6
**Type of malignancy**					
Breast cancer	10	4	1	–	15
Cervical cancer	9		4	1	14
Colorectal cancer	4	–	3	1	8
Thyroid cancer	1	5	1	1	8
Lung cancer	5	–	2	–	7
Endometrial cancer	1	2	1	–	4
Prostatic cancer	3	–	–	1	4
Renal cancer	2	1	–	–	3
Basalioma	–	–	3	–	3
Leukaemia	2	1	–	–	3
Neuroendocrine cancer	2	–	1		3
Other cancers	7	9	4	–	20
Total	46 (in 43 patients)	22 (in 21 patients)	20	4	92 (in 88 patients)

Mg, malignancy; PA, pituitary adenoma.

A recently published study in patients with non-functioning pituitary adenoma analyzed chromosomal and oxidative DNA damage (Bitgen *et al.* 2021). These authors reported increased chromosomal DNA damage parameters in these patients, which might be associated with possible cancer risk in these patients. Similar results of increased genome damage were published in acromegaly and prolactinoma (Hamurcu *et al.* 2011, Bayram *et al.* 2014, Bitgen *et al.* 2016).

We recently encountered a 57-year-old male patient with a background history of breast cancer who presented with visual disturbance. He was initially diagnosed with a ductal breast carcinoma 2 years prior and was treated with mastectomy. Post-operatively, he was commenced on tamoxifen. Consecutively he was diagnosed with a non-functioning silent gonadotroph pituitary tumor. His mother suffered from breast cancer, and because of the genetic risk, he was evaluated for breast cancer gene 1 (BRCA1)/breast cancer gene 2 (BRCA2) mutation and was positive.

There are 21 reported cases and series in the literature on metastasis of malignancy to a pituitary non-secreting-gonadotroph adenoma, reminding us of the coexistence of two diagnoses and the need for the correct solution of the relationship (Barbosa *et al.* 2020).

### Prolactinoma and risk for malignancy

Prolactinoma is a very common type of pituitary tumor. There are experimental data indicating that prolactin might play a role in progression and invasion of several cancer types, especially breast cancer (Clevenger *et al.* 2003, Liby *et al.* 2003)*.*Several case reports of breast cancer in both females and males with prolactinoma confirm experimental data (Forloni *et al.* 2001, Sato *et al.* 2007, Mallawaarachchi *et al.* 2011, Zheng *et al.* 2017, Bettencourt-Silva *et al.* 2018, Hao *et al.* 2020). Both diagnoses were either diagnosed simultaneously or in the majority, with a significant latency period up to 32–36 years questioning the role of hyperprolactinemia in inducing cancer. Epidemiological studies that have examined the relationship between prolactin levels and all-cause cancer and/or breast cancer reported inconsistent results (Tworoger *et al.* 2006, 2013, Dekkers *et al.* 2010, 2015, Berinder *et al.* 2011, Tikk *et al.* 2015, Wang *et al.* 2016, Soto-Pedre *et al.* 2017, Pekic *et al.* 2019*a*).

A population-based cohort study that investigated cancer risk in patients with hyperprolactinemia reported a small increase in overall cancer risk, mainly attributed to upper gastrointestinal cancer and hematopoietic cancer, but the risk of breast cancer was not increased, and the risk of prostate cancer was reduced (Berinder *et al.* 2011). A large proportion of patients with breast cancer have a family history of breast or ovarian cancer. The most common gene mutations associated with breast cancer are BRCA1 and BRCA2 mutations. Bettencourt-Silva *et al*. reported a case of a 25-year-old female with giant prolactinoma, germline BRCA1 mutation and a family history of breast cancer (mother and maternal aunt) (Bettencourt-Silva *et al.* 2018). This case is in line with our data that there is a strong association between prolactinoma and family history of breast cancer (Table 3) (Pekic *et al.* 2019*b*).

**Table 3:** Clinical characteristics and history of malignancy in patients with pituitary adenoma and their families. Reprinted by permission from Springer Nature Ltd., Pekic S, Soldatovic I, Miljic D, Stojanovic M, Doknic M, Petakov M & Popovic V, *Hormones and Cancer*, Familial cancer clustering in patients with prolactinoma, © 2019.

	Acromegaly	Prolactinoma	NFPA	Total	*P*
*n*	220	372	470	1062	
Male sex (%)	86 (39.1)^p^	96 (25.8)^a, n^	204 (43.4)^p^	386 (36.3)	0.001
Age (mean ± s.d., years)					
Actual age	54.9 ± 13.5^p^	41.1 ± 13.8^a, n^	55.5 ± 16.5^p^	50.6 ± 16.5	0.001
At diagnosis of PA	47.2 ± 13.6^p, n^	35.4 ± 13.0^a, n^	51.2 ± 16.1^a, p^	44.9 ± 16.2	0.001
Malignancy in the family (%)	96 (43.6)	192 (51.6)^n, b^	207 (44.0)^p, b^	495 (46.6)	0.056
1° rel	55 (25.0)	77 (20.7)^n^	140 (29.8)^p^	272 (25.6)	0.011
Parents	41 (18.6)	66 (17.7)^n, b^	110 (23.4)^p, b^	217 (20.4)	0.098
Siblings	15 (6.8)^p^	11 (3.0)^a,n^	34 (7.2)^p^	60 (5.6)	0.020
2 and 3° rel	55 (25.0)^p^	144 (38.7)^a,n^	99 (21.1)^p^	298 (28.1)	0.001
All degrees rel^c^	14 (6.4)	29 (7.8)	32 (6.8)	75 (7.1)	0.673
Breast cancer in 1° rel (%)	4 (1.8)^n, p, b^	19 (5.1)^a^	29 (6.2)^a, b^	52 (4.9)	0.046
Lung cancer in 1° rel (%)	9 (4.1)	9 (2.4)	16 (3.4)	34 (3.2)	0.507
Colon cancer in 1° rel (%)	6 (2.7)	13 (3.5)	16 (3.4)	35 (3.3)	0.866
Breast cancer in 2 and 3° rel (%)	12 (5.5)^p^	41 (11.0)^a, n^	19 (4.0)^p^	72 (6.8)	0.001
Lung cancer in 2 and 3° rel (%)	14 (6.4)	37 (9.9)^n^	18 (3.8)^p^	69 (6.5)	0.002
Colon cancer in 2 and 3° rel (%)	5 (2.3)	21 (5.6)	25 (5.3)	51 (4.8)	0.140

Single group by group comparisons, with correction (*P*  < 0.05): ^a^Acromegaly; ^p^Prolactinoma; ^n^NFPA; ^b^Single group by group comparisons, without correction (*P*  < 0.05); ^c^Some patients are represented in more than one category if having both a first-degree and second- and third-degree relatives with a cancer.

1°, first-degree relatives; 2 and 3°, second- and third-degree relatives; NFPA, non-functioning pituitary adenoma; PA, pituitary adenoma; rel, relatives.

### Prolactinoma in two patients with uncommon disease

We have previously reported two patients who were found to have two rare conditions: one male patient had developmental hypothalamic–pituitary disorder: Kalman syndrome (KS) and the other female patient had congenital hypopituitarism and both were diagnosed with a pituitary tumor (prolactinoma) in adulthood (Doknic *et al.* 2012).

A male patient with hypogonadotropic hypogonadism – КS due to KAL 2 gene mutation (loss of function of fibroblast growth factor receptor 1 – FGF R 1 mutation) and a female patient with congenital hypopituitarism (combined pituitary hormone deficiency - CPHD) due to PROP1 (pituitary homeobox protein prophet of the Pit 1 gene mutation) who both developed in adulthood well-differentiated pituitary adenoma – prolactinoma. The latency period before the diagnosis of prolactinoma for the male patient with KS was 25 years, while for the female patient with PROP1 mutation was 10 years.

To our knowledge, there is only one recent report by Suzuki *et al*. (2021) who showed that the PIT-1β mutation can cause CPHD through a novel genetic mechanism, such as PIT-1β overexpression, and that POU1F1 mutation might be associated with a prolactinoma (Suzuki *et al.* 2021)*.*These authors state that it is unclear how the PIT-1β mutation is associated with the development of prolactinoma and that there is no clear explanation regarding the possible mechanism for the association between developmental hypothalamic–pituitary disorders with the development of pituitary tumors. Are they separated and potentially unrelated disease processes that may occur concomitantly or not is to be shown in further studies We should not dismiss reports that mutations in early developmental transcription factor such as SOX2 are associated with slowly progressing hypothalamo–pituitary tumor (Alatzoglou *et al.* 2011). Also, it has been shown recently that activating mutations in BRAF disrupt the hypothalamo–pituitary axis development leading to hypopituitarism, while somatic mutation of BRAF pV600E is a driver of benign tumor, papillary craniopharyngioma and is identified in corticotroph adenomas (Gualtieri *et al.* 2021). Mutations in BRAF have a high occurrence rate in different cancers. So the same underlying genetic driver has different unknown roles in different cell types leading to tumorigenesis (Gualtieri *et al.* 2021).

Occam razor principle is to ‘choose the simplest from a set of models of a given phenomenon that is in accordance with evidence’ (Wildner 1999)*.*The patient with PROP1 mutation in her family had an occurrence of multiple malignancies such as breast cancer, melanoma and lung cancers while the mother of the patient with KS suffered from breast cancer.

## Studies on familial cancer risk in patients with pituitary adenoma

The association of pituitary adenoma and positive family cancer history has been demonstrated in few studies (Hemminki *et al.* 2007, Couldwell & Cannon-Albright 2014, Terzolo *et al.* 2017, Pekic *et al.* 2019*b*). The epidemiological study on incidence and familial risks in pituitary adenoma and associated tumors has been reported in the Swedish Family-Cancer Database of 10.5 million individuals containing families with parents and offspring, included 3239 patients with pituitary tumor (Hemminki *et al.* 2007). Hemminki *et al*. reported that among tumors associated with pituitary adenoma in families, breast and colorectal cancer were prominent. Second large epidemiological study analyzed the Utah Population Database with more than 7.5 million individuals, including 575 patients with pituitary adenoma (Couldwell & Cannon-Albright 2014). The analysis of this database showed an association of pituitary adenoma with excess of several cancers (prostate and other cancer sites) among their relatives. Our large study on 1062 patients with pituitary adenomas showed that family history of malignancy should not be dismissed as a risk for pituitary adenoma development (Pekic *et al.* 2019*b*). We demonstrated that almost half of patients (46.6%) with pituitary adenoma of any type (prolactinoma, non-functioning pituitary adenoma and acromegaly) had a family history of malignancy (Table 3) (Pekic *et al.* 2019*b*). In our series, we demonstrated a strong association between prolactinoma and family history of breast and lung cancers. Also, colorectal cancer was associated with all types of pituitary adenomas. An interesting observation in our study was that younger age and female sex were more prevalent in pituitary adenoma patients with positive family cancer history (Pekic *et al.* 2019*b*).

The importance of detailed family history is reported in recently published 2021 National Comprehensive Cancer Network Guidelines principles of genetic risk assessment and counseling in patients with sporadic and hereditary neuroendocrine and adrenal tumors (Shah *et al.* 2021). These recommendations included in pre-test counseling detailed personal cancer/tumor history (including age at diagnosis and treatment) and detailed family history (including cancers/tumors and age at diagnosis, as well as clinical symptoms that can indicate underlying endocrine neoplasia) in first-, second- and third-degree family members on each side of the family (Shah *et al.* 2021).

## Genomic profile of pituitary tumors

Accumulation of genetic alterations is involved in cancer pathogenesis. Various cancers use different molecular pathways. Today, it is recognized that cancer evolves in a stepwise fashion and it may take decades to progress (Shain *et al.* 2015)*.*It is also believed that the accumulation of genetic defects results in monoclonal expansion of pituitary adenomas. Most pituitary tumors are aneuploid suggesting genetic instability. Analysis of pituitary adenomas using whole-exome sequencing shows sporadic mutations at relatively low prevalence (Bi *et al.* 2017). In that study, genomic profile of a large cohort of pituitary tumors demonstrated high variability in somatic copy number profiles and genomic disruption. None of the molecular studies could identify critical molecular signatures associated with pituitary tumorigenesis. What is identified are frequent changes in genes in the DNA damage response pathway, cell cycle and chromatic modifier families. Some somatic mutations, like *GNAS* gain-of-function mutation identified in 30% of somatotropinomas, *GPR101* mutation identified in 4% of somatotropinomas and *USP8* mutation identified in 40% of corticotrophinomas, are not clearly associated with tumor behavior (Reincke *et al.* 2015, Chen *et al.* 2018, Sbiera *et al.* 2019). Some pituitary adenomas express no chromosomal alterations, while the other has widespread genomic disruptions, functioning pituitary adenomas in particular (Bi *et al.* 2018, Hage *et al.* 2018). Widespread genomic alterations are present in silent corticotroph adenoma and prolactinoma in particular (Bi *et al.* 2017).

The ability to accurately identify molecular signatures associated with histopathology and clinical outcomes is still not possible. Although a study by Lasolle *et al*. did show that the quantity of copy number variations is dependent on tumor type (higher in prolactinomas) compared to other tumors and another study showed that all functional pituitary tumors demonstrated higher variability in copy number profiles (Bi *et al.* 2017, Lasolle *et al.* 2020)*.*As for aggressive and malignant pituitary tumors, lactotroph tumors are the second most frequent after corticotrophinomas (McCormack *et al.* 2018). Gene expression analysis of lactotroph tumors showed significant deregulation of 140 genes (120 genes showed increased expression, while 20 genes showed reduced expression) (Wierinckx *et al.* 2018). There is a sex-specific gene landscape, with some genes expressed at higher levels in males compared with females: (1) two growth factors, *FGF13*and *VEGFD* involved in angiogenesis, cell growth/proliferation and control of cellular movement/morphology, *VEGFD* involved also in cell cycle control; (2) cancer-testis antigen (*CTAG2*) involved in cellular movement, highly expressed also in invasive breast cancer; (3) creatine transporter (*SLC6A8*) involved in creatine metabolism (Wierinckx *et al.* 2018). These findings could explain sex-specific differences in lactotroph adenoma behavior (men have a higher grade of lactotroph adenoma, more resistance to treatment, worse prognosis). These authors also observed that chromosomic abnormalities were numerous in aggressive than non-aggressive tumors, implying much pronounced genetic instability and tumor aggressiveness. Another important finding of these authors is a reduced estrogen receptor alpha (ERα) protein expression in male lactotroph tumors (Delgrange *et al.* 2015, Wierinckx *et al.* 2018, Trouillas *et al.* 2019). It is well-known that a correlation between low ERα protein expression in breast tumor and the grade of malignancy exists (Krol *et al.* 2018).

Recently, a multi-omics analysis of 134 pituitary tumors identified chromosomal alterations in 23% of the genome been altered (Neou *et al.* 2020). Again, the number of alterations was higher in functional tumors. This integrated pangenomic analysis of pituitary tumors identified different molecular classes of these tumors allowing a new, pangenomic classification with new subtypes, with implications for the clinicians.

An investigation of telomere length in pituitary adenoma was recently published (Heaphy *et al.* 2020). Shortened, dysfunctional telomeres are often detected in precancerous lesions and invasive cancers, contributing to genomic instability. The study of 106 pituitary adenomas demonstrated that short telomere lengths were detected in half of the cases (59.4%) (Heaphy *et al.* 2020). Also, 36% of recurrent pituitary adenomas had alternative lengthening of telomeres. The authors concluded that telomere alterations are prevalent in pituitary adenomas, contributing to genomic instability (Heaphy *et al.* 2020). As has been reported many years ago, it still remains uncertain whether the molecular defects that have been found within the pituitary tumor are acquired and if they are pathogenic (Levy & Lightman 2003, Levy 2011).

In summary, the accumulation of genetic alterations is involved both in pituitary tumor and cancer pathogenesis.

## Environmental risk factors

Age, family history and hereditary cancer predisposition syndromes present non-modifiable cancer risk factors. However environmental insults are modifiable, that is avoidable cancer risk factors. Metabolic disturbances (obesity and diabetes mellitus) will overtake smoking and alcohol as modifiable cancer risk factors. ‘Insulin resistance’ (observed in obesity and type 2 diabetes mellitus) does not apply equally to all of insulin’s actions. Adverse trophic actions of the high insulin concentration in insulin resistance required to maintain euglycemia have been reported. These trophic actions of increased insulin actions in different tissues are in particular observed in ovaries in patients with polycystic ovary syndrome (Huang-Doran *et al.* 2021).

Environmental risk factors were long accepted as contributors to increased risk of several malignancies. More recently, a considerable scientific interest was directed toward environmental pollutants and endocrine disruptor chemicals as risk factors in pituitary adenoma pathogenesis or promotion. The most investigated possible crossroad for environmental toxins (dioxins and heavy metals) and pituitary tumorigenesis is the AIP–AhR pathway. Scientific interest for the nature and function of *AIP* gene (Aryl hydrocarbon receptor-interacting protein) originated from the discoveries that inactivating germline *AIP* mutations are associated with occurrence of pituitary adenomas (Alforei & Korbonits 2014, Lim & Korbonits 2018). Inactivating *AIP* mutations lead to pituitary adenoma in 30%, most frequently to somatotropinoma or prolactinoma, often large, invasive and resistant to medical treatment and prone to apoplexy (Vierimaa *et al.* 2006, Leontiou *et al.* 2008, Trivellin & Korbonits 2011, Lloyd & Grossman 2014, Pepe *et al.* 2019). Frequent invasiveness and clinical aggressiveness of *AIP* mutation-associated pituitary adenoma are in contrast to other monogenetically predisposed pituitary adenoma promoted by the cAMP–PKA pathway activation (such as Carney complex or McCune–Albright syndrome). This suggests different or additional mechanisms in the case of *AIP* (Pepe *et al.* 2019). Pituitary adenoma in *AIP* mutation carriers typically occurs with a low penetrance (Vierimaa *et al.* 2006, Chahal *et al.* 2012). This may also indicate that additional environmental or genetical factors might contribute to the risk of pituitary adenoma development in *AIP* mutation carriers. The first identified partner molecule of AIP protein was a nuclear receptor – AhR, best known for its role in binding environmental toxins. Thus, environmental pollution was investigated as one of the possible factors in pituitary adenoma pathogenesis or promotion via AIP–AhR pathway. This problem was investigated *in vitro* on pituitary cell lines and in acromegaly patients (Pesatori *et al.* 2008, Cannavò *et al.* 2010, Cannavo *et al.* 2016, 2017, Fortunati *et al.* 2017, Tapella *et al.* 2017). Several *in vitro* studies demonstrated a relationship between GH3 cells exposure to toxins acting via AhR and an increase in proliferation (Tapella *et al.* 2017). On an epidemiological level, a correlation was suggested between exposure to environmental air contaminants and increase in somatotropinoma incidence (Cannavò *et al.* 2010). However, a direct relationship was not confirmed by investigating the population in a region affected by a major industrial accident with widespread dioxin exposure (Pesatori *et al.* 2008). A modifying effect of *AhR* or *AIP* polymorphisms in addition to contaminant exposure was associated in acromegaly patients with pituitary adenoma size, biological aggressiveness and resistance to somatostatin analogs (Cannavo *et al.* 2016).

Cadmium is a heavy metal of considerable environmental and occupational concern. Several regulatory agencies classified cadmium compounds as human carcinogens, based on the studies indicating occupational cadmium exposure association with lung cancer. A waker linkage was postulated between cadmium exposure and human prostate and renal cancer (Waalkes *et al.* 1999). Meanwhile, an association was demonstrated between a single, high-dose exposure to cadmium in Noble rats with a significant increase in pituitary adenoma prevalence, exclusively in pars distalis (Waalkes *et al.* 1999). The effects of cadmium on pituitary tumorigenesis need further elucidation but might also involve the *AhR-AIP* pathway since AhR signaling was recently observed to be involved in the lung leukocyte proinflammatory cytokine response to cadmium (Kulas *et al.* 2021).

Bisphenol A (BPA) is a monomer of plastics and epoxy resins widely used in the dentistry and food packaging industry, persistently present in the environment due to its long biological half-life. BPA exposure was identified to present a potential risk factor for breast cancer (Keshavarz-Maleki *et al.* 2021). On the other hand, high BPA concentrations induce cell growth and prolactin secretion in an estrogen-responsive pituitary tumor cell line (Maruyama *et al.* 1999, Chun & Gorski 2000).

Polychlorinated biphenyls (PCBs) are persistent pollutants and pro-tumorigenic in the liver. Although non-dioxin like, these substances were also observed to affect the pituitary AhR pathway. Data on the capacity of PCBs to modulate cell proliferation at the pituitary level signal a potential tumor-promoting role of this pollutant (Raggi *et al.* 2016).

Endocrine-disrupting chemicals (EDCs) are low-concentration pollutants from various sources capable of mimicking hormonal actions or affecting endocrine pathways, by interfering with the synthesis, metabolism, binding or cellular responses of natural hormones (Hamid *et al.* 2021). EDCs have been associated with cancer prevalence, especially breast, prostate and testicular malignancies (Rocha *et al.* 2021). Xeno-estrogens are one of major EDC classes, attributed to genomic or non-genomic interactions with estrogen receptor. Xeno-estrogens, such as alkylphenols (in particular, nonylphenol) are found to have a proliferative effect on pituitary level (Kochukov *et al.* 2009). The capability of natural estrogens to influence pituitary tumorigenesis was only observed in higher doses. Thus, weak estrogenic EDCs are unlikely to cause tumorigenesis alone but are more likely to promote the growth of existing pituitary tumors (Fujimoto 2001).

## Conclusion

Data show that dual diagnoses do arise: common (cancers) and uncommon tumors (pituitary tumors) in the same patient. Following the Occam razor principle, we believe that strong family history of malignancy (inherited trophic mechanisms) in our patients in the context of unfavorable environment is the underlying risk factors for the development of cancer and pituitary tumorigenesis.

## Declaration of interest

The authors declare that there is no conflict of interest that could be perceived as prejudicing the impartiality of this review.

## Funding

This study was supported by a grant from the Ministry of Science of Republic of Serbia (Project 175033).

## Author contribution statement

S P, M S and V P wrote the paper.
